# Urine colorimetry to detect Low rifampin exposure during tuberculosis therapy: a proof-of-concept study

**DOI:** 10.1186/s12879-016-1576-1

**Published:** 2016-06-01

**Authors:** Isaac Zentner, Hans P. Schlecht, Lorna Khensouvann, Neo Tamuhla, Michele Kutzler, Vijay Ivaturi, Jotam G. Pasipanodya, Tawanda Gumbo, Charles A. Peloquin, Gregory P. Bisson, Christopher Vinnard

**Affiliations:** Department of Biochemistry and Molecular Biology, Drexel University College of Medicine, Philadelphia, PA USA; Department of Medicine, Division of Infectious Diseases, Drexel University College of Medicine, Philadelphia, PA USA; Genomind, Inc, King of Prussia, PA USA; Botswana-Upenn Partnership, Gaborone, Botswana; Center for Translational Medicine, School of Pharmacy, University of Maryland, Baltimore, MD USA; Center for Infectious Disease Research and Experimental Therapeutics, Baylor Research Institute, Baylor University Medical Center, Dallas, TX USA; College of Pharmacy and Emerging Pathogens Institute, University of Florida, Gainesville, FL USA; Department of Medicine, Division of Infectious Disease, Perelman School of Medicine at the University of Pennsylvania, Philadelphia, PA USA; Public Health Research Institute, New Jersey Medical School, Rutgers University, Newark, USA

**Keywords:** Tuberculosis, Point-of-care, Therapeutic drug monitoring

## Abstract

**Background:**

The cost and complexity of current approaches to therapeutic drug monitoring during tuberculosis (TB) therapy limits widespread use in areas of greatest need. We sought to determine whether urine colorimetry could have a novel application as a form of therapeutic drug monitoring during anti-TB therapy.

**Methods:**

Among healthy volunteers, we evaluated 3 dose sizes of rifampin (150 mg, 300 mg, and 600 mg), performed intensive pharmacokinetic sampling, and collected a timed urine void at 4 h post-dosing. The absorbance peak at 475 nm was measured after rifamycin extraction. The optimal cutoff was evaluated in a study of 39 HIV/TB patients undergoing TB treatment in Botswana.

**Results:**

In the derivation study, a urine colorimetric assay value of 4.0 × 10^−2^ Abs, using a timed void 4 h after dosing, demonstrated a sensitivity of 92 % and specificity of 60 % to detect a peak rifampin concentration (C_max_) under 8 mg/L, with an area under the ROC curve of 0.92. In the validation study, this cutoff was specific (100 %) but insensitive (28 %). We observed similar test characteristics for a target C_max_ target of 6.6 mg/L, and a target area under the drug concentration-versus-time curve (AUC_0–8_) target of 24.1 mg•hour/L.

**Conclusions:**

The urine colorimetric assay was specific but insensitive to detect low rifampin serum concentrations among HIV/TB patients. In future work we will attempt to optimize sampling times and assay performance, with the goal of delivering a method that can translate into a point-of-care assessment of rifampin exposure during anti-TB therapy.

Standardized anti-tuberculosis (TB) drug regimens that include rifampin, isoniazid, pyrazinamide, and ethambutol are the foundation of the global public health response to the TB epidemic [1]. Yet there is wide variability in absorption and metabolism of the anti-TB drugs, and low drug concentrations in blood are associated with inferior TB treatment outcomes, including treatment failure and relapse [2–5]. Pharmacokinetic variability has been identified as a key mediator of the rate of sterilizing effect and the emergence of new drug resistance mutations during anti-TB therapy [6, 7].

The TB clinician must consider the totality of the clinical information when making therapeutic decisions regarding dose adjustment. When therapeutic drug monitoring is performed, the result (a concentration above or below a threshold value) would support a clinical decision to increase the dose size of one or more of the drugs. The impact of this decision is evaluated during the clinical course, sometimes with repeated monitoring to ensure that the therapeutic target has been reached [8]. While the output from therapeutic drug monitoring provides a continuous measure of drug concentration, the practical use of that information is to classify the patient as having adequate or inadequate drug exposure.

In resource-rich settings, therapeutic drug monitoring can be performed during anti-TB therapy by measuring plasma or serum drug concentrations. The clinical decision to perform therapeutic drug monitoring may be motivated by factors such as a slow treatment response, the need for second-line drugs during the treatment of multi-drug resistant infection, or the presence of co-morbidities associated with inferior treatment outcomes (HIV co-infection, diabetes mellitus) [9]. The performance of therapeutic drug monitoring requires specialty laboratory capabilities, such as high performance liquid chromatography (HPLC) or gas chromatography (GC). Technical expertise is also required to collect, process, and ship samples to the specialty laboratory.

In many high-burden settings, the complexity and cost of these laboratory methods may seem to preclude the use of therapeutic drug monitoring in the clinical care of TB patients. Consequently, patients with inadequate drug exposure cannot be identified early during anti-TB therapy, at a time when dosing adjustments could be expected to improve treatment outcomes. Although dried blood spots have been proposed as a means to facilitate sample collection in the field [10], the measurement of drug concentrations in whole blood using dried blood spot techniques requires the same laboratory capabilities as traditional methods, and the interpretation of drug concentrations in whole blood (versus serum or plasma) is uncertain [11].

A simple, inexpensive test to classify TB patients based on an estimate of drug exposure, available at the point-of-care at the time of the patient encounter, could supplement other clinical information in support of treatment decisions. Urine colorimetry was first evaluated in the 1970’s as a method to assess the bioequivalence of different fixed-dose combinations of anti-TB drugs [12–14]. More recently, a urine colorimetric approach to detect isoniazid in urine (the “Arkansas method”) has been commercialized as a tool to monitor isoniazid adherence (IsoScreen, GFC Diagnostics LTD, Oxfordshire, UK) [15]. Potential advantages of urine colorimetric methods include a non-invasive sampling approach, improved patient acceptability, and the low cost and stability of chemical reagents.

We sought to determine whether urine colorimetry could have a novel application as a measure of systemic rifampin exposure during anti-TB therapy, as defined by the maximum serum concentration (C_max_) or the area under the concentration-versus-time curve (AUC_0–8_). Our approach was first to develop the urine colorimetric assay among healthy volunteers, and then to validate the assay among HIV/TB patients from a high-burden setting.

## Methods

### Derivation study

#### Study design

We performed a non-randomized, open-label, cross-over study of the first-line anti-TB drugs (rifampin, isoniazid, ethambutol, pyrazinamide) in 6 healthy volunteers. We sequentially evaluated 3 dose sizes in separate study visits, with rifampin dosed at 150 mg, 300 mg, and 600 mg. Each study visit was separated by a wash-out period of at least 1 week. Blood samples were collected prior to oral administration of the study drugs, and then at 1, 2, 4, 6, and 8 h following oral administration of the study drugs. Frozen samples were shipped to the Infectious Disease Pharmacokinetics Laboratory at the University of Florida for measurement of serum drug concentrations. All urine was collected during the study visit, and the volume and time of collection were noted. Timed voids were obtained at 4 h and 8 h post-dosing. Urine was aliquoted into single-use 3 mL conical vials and stored at -70C until ready for analysis.

#### Urine colorimetric assay

We followed the Sunahara method to extract total rifamycins from urine samples [16]. In brief, 50 μl of 100 mM phosphate buffer (pH 7) was added to 100 μl of urine sample followed by 100 μl of isoamylalcohol. Each sample was mixed by vortexing for 20 s at max speed. Samples were centrifuged at 14,000 rpms for 5 min at room temperature. The aqueous phase (upper) was carefully removed and transferred to a clear 96-well plate and optical density was measured at 475 nm in a Multiskan™ GO Microplate Spectrophotometer (Thermo Fisher Scientific). A calibration curve was determined for 10 serial dilutions (1:2) starting at 1000 mg/L of pure rifampin (Sigma Aldrich) that had been extracted with isoamylalcohol.

#### Statistical analysis

The goal of the statistical analysis for the development cohort was to define the accuracy of the urine colorimetric assay to detect low rifampin serum C_max_ or AUC_0–8_, across a range of possible cutoff values, and to identify the optimal cutoff value for subsequent evaluation in the validation cohort. The area under the receiver-operating-characteristic (ROC) curve provides a summary measure of the ability of the diagnostic test to distinguish between adequate and inadequate rifampin exposure. An area under the ROC curve equal to 1 demonstrates perfect discrimination, whereas an area of 0.5 demonstrates that the diagnostic test performs no better than chance alone [17].

For our primary analysis, we evaluated the ROC curve for the urine colorimetric assay corresponding to a serum rifampin C_max_ target of 8 mg/L, which is the standard-of-care target for therapeutic drug monitoring during anti-TB treatment [8]. The 95 % confidence interval for the area under the ROC curve was calculated using 2000 bootstrap replicates [18]. In an a priori decision, we defined the optimal cutoff for the urine colorimetric assay as the value corresponding to 90 % sensitivity for each drug exposure target. Statistical significance was declared for p-values less than 0.05. All statistical analysis was performed in R, with non-compartmental pharmacokinetic (PK) analysis performed using the *PK* package, and ROC analysis performed using the *pROC* package [19].

### Validation study

#### Setting and participants

The validation study was nested within a prospective cohort study of anti-TB drug PK in HIV/TB patients at 22 public clinics and Princess Marina Hospital in Gaborone, Botswana. HIV-infected adults (21 years of age and older) were eligible for enrollment in the parent study if they were citizens of Botswana, naïve to antiretroviral therapy (ART), and newly diagnosed with pulmonary TB. Patients must have been initiated on a standard first-line TB treatment regimen, following WHO guidelines for weight-based dosing bands. The diagnosis of pulmonary TB must have been established by either a positive sputum smear, a positive GeneXpert MTB/RIF assay (Cepheid, Sunnyvale, CA, USA), or the presence of WHO criteria for smear-negative pulmonary TB. Exclusion criteria included pregnancy, renal insufficiency (defined as a creatinine clearance less than 50 mL/min, and hepatic dysfunction (defined as either an alanine transaminase or aspartate transaminase greater than 3 times the upper limit of normal).

#### Procedures

The PK study visit was scheduled prior to the completion of the intensive phase of anti-TB therapy. All PK visits were conducted at the Infectious Disease Care Clinic at Princess Marina Hospital. Oral doses of the anti-TB drugs were obtained from the Gaborone City Clinic and directly administered to the participant on the morning of the PK visit. A baseline blood sample was drawn prior to dosing, and then at 0.3, 0.9, 2.2, 4.5, and 8 h post-dosing. These sampling times were selected based on the objectives of the parent study to evaluate the population PK of isoniazid. At each time point, 10 mL of blood was drawn and transported to the Botswana Harvard Partnership Laboratory. After centrifugation, serum was stored at -70C. Serum drug concentrations were measured at the Gumbo Laboratory at the Baylor Research Institute (Dallas, TX) using liquid chromatography-tandem mass spectrometry methods. For the performance of the urine colorimetric assay, a single urine sample was obtained 4 h after dosing, based on the diagnostic accuracy of this time point to identify healthy subjects with C_max_ less than 8 mg/L in the derivation study. Urine samples were frozen and shipped to the Infectious Disease Clinical Research Laboratory at Drexel University College of Medicine (Philadelphia, PA). The urine assay steps were performed as described for the healthy volunteers.

#### Statistical analysis

We evaluated the overall distribution of the urine colorimetric assay in the validation sample, along with potentially relevant covariates (age, body weight, renal function). We plotted individual rifampin concentration-versus-time profiles for each patient, and identified the corresponding rifampin C_max_ for each patient. Non-compartmental analysis was performed to estimate rifampin AUC_0–8_ for each patient that completed the study visit.

Validation of the urine colorimetric assay was performed for 3 separate targets. In the primary analysis, we validated the optimal cutoff previously identified from the healthy subjects to detect C_max_ less than 8 mg/L [8]. In secondary analyses, we evaluated two additional drug exposure targets. A C_max_ target of 6.6 mg/L was recently shown to predict delayed sputum conversion in a South African cohort [3]. Based on pre-clinical rifampin pharmacodynamic data, we also evaluated a rifampin AUC_0–8_ target of 24.1 mg•hour/L [20]. For each target, bootstrapping (2000 replicates) was performed to estimate the 95 % confidence interval for the area under the ROC curve [18]. We assessed sensitivity, specificity, positive and negative predictive values, and positive and negative likelihood ratios at the cutoff identified in the derivation study. We also calculated 90 % confidence intervals for sensitivity and specificity with 2000 bootstrap replicates [18].

## Results

### Derivation study

**Fig. 1:**
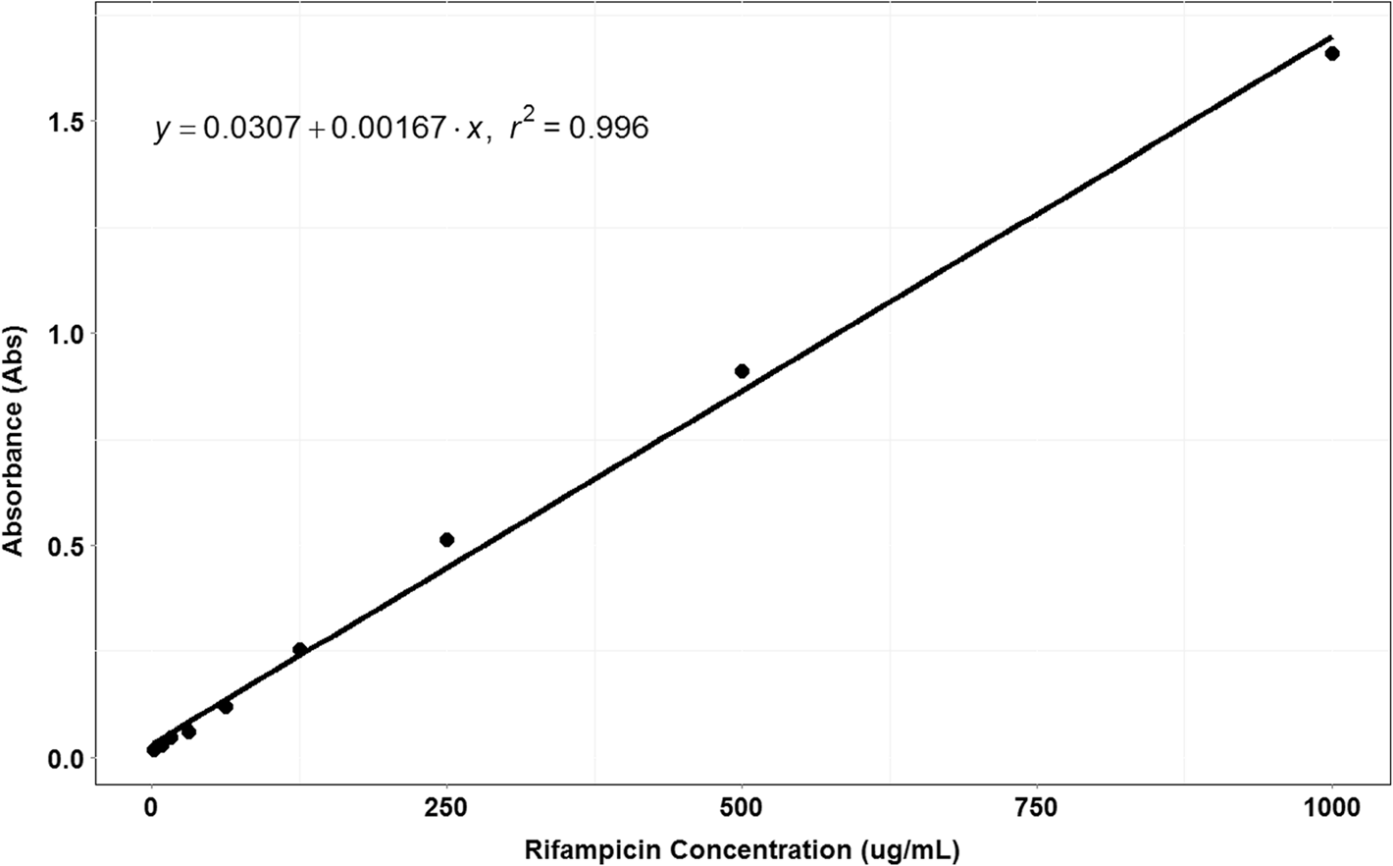
Calibration curve for the urine colorimetric assay

Calibration of the urine colorimetric assay with known standards is shown in Fig. 1. The extraction of rifampin via Sunahara method demonstrated a linear relationship between the absorbance peak at 475 nm and standard rifampin concentrations in a range from 2.0 to 1000 mg/L (R^2^ greater than 0.99), which is similar to the original report.

**Fig. 2:**
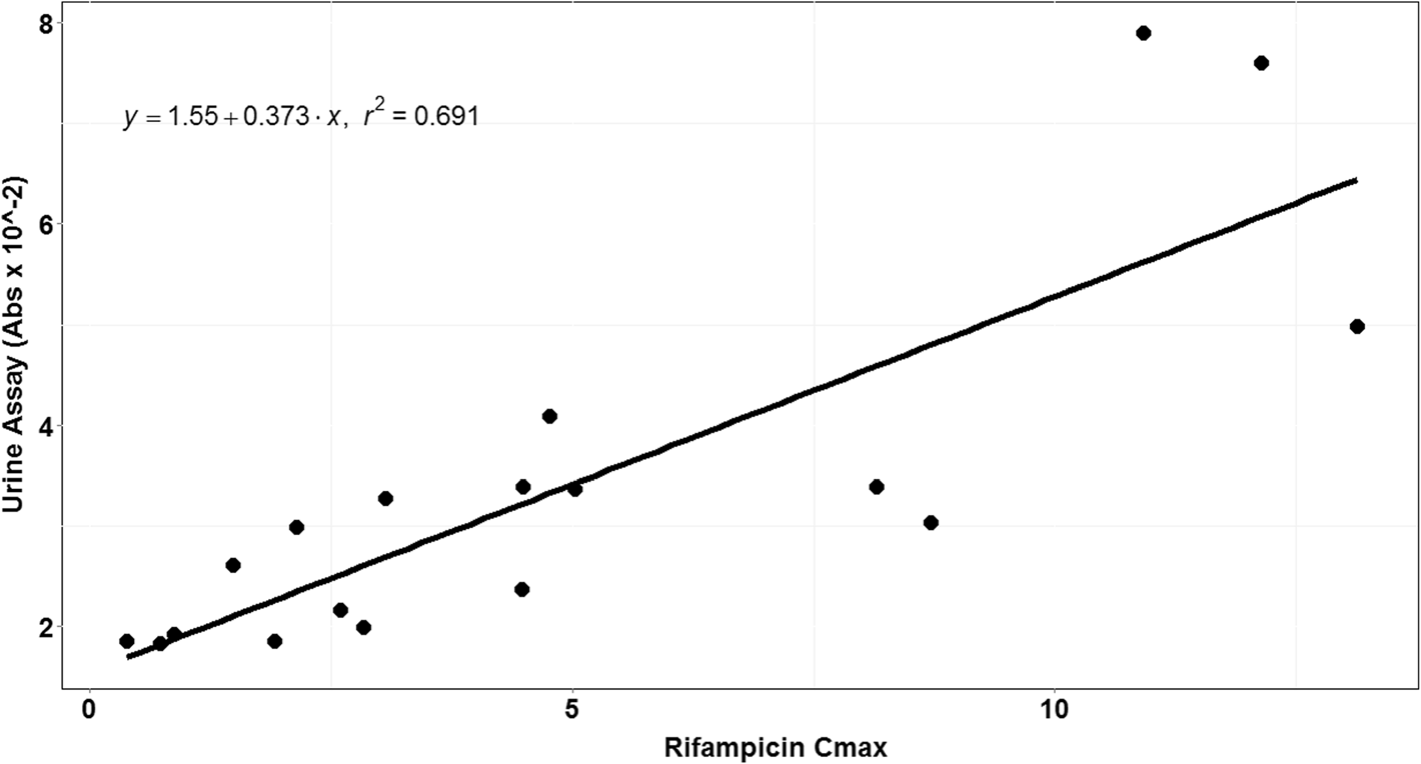
Correlation between urine colorimetric assay and serum rifampin C_max_ among healthy volunteers

**Fig. 3:**
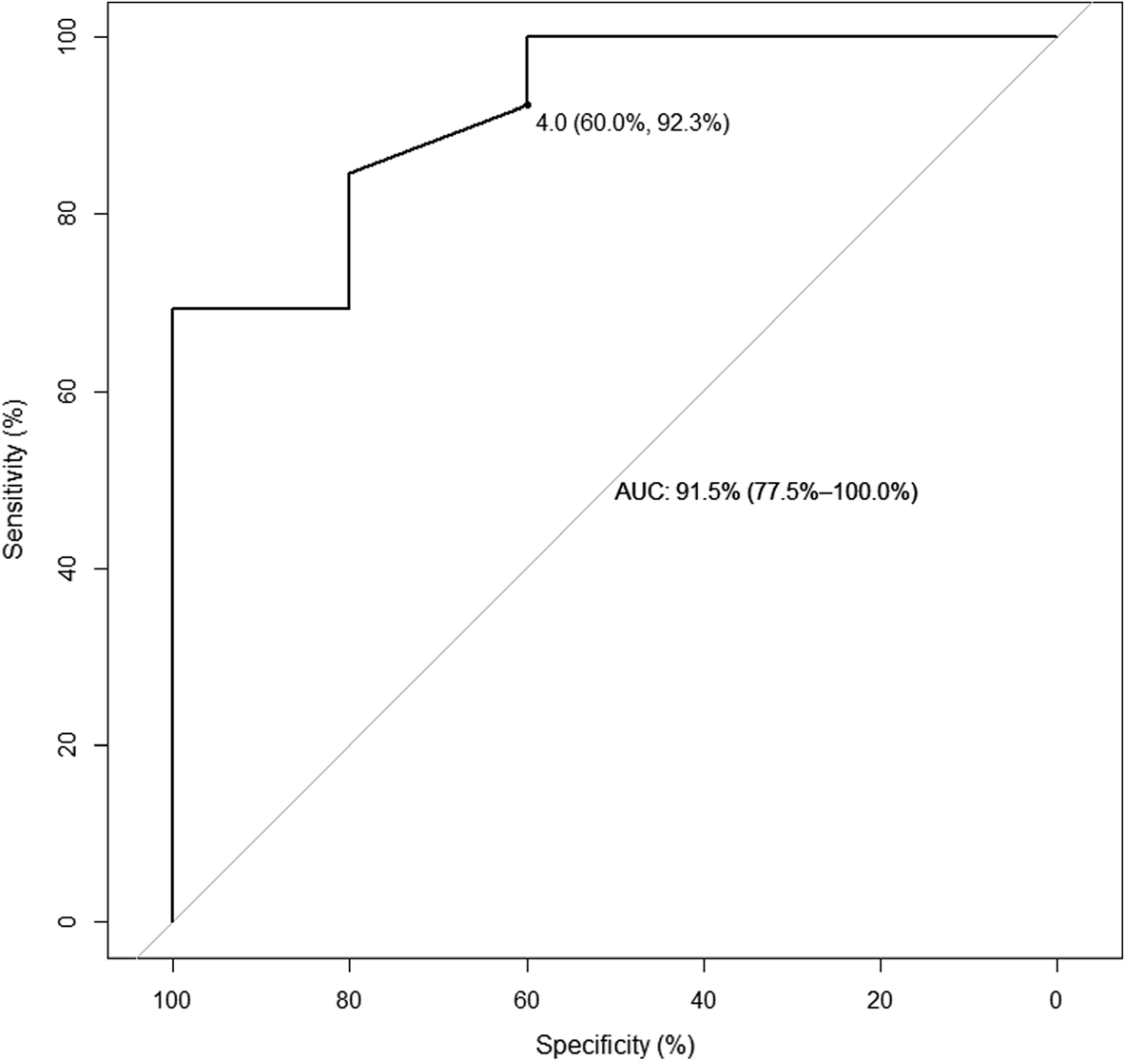
ROC analysis for urine colorimetric assay to detect rifampin C_max_ less than 8 mg/L

We enrolled 6 healthy volunteers in the derivation study, and each volunteer completed all study visits. Delayed oral absorption of rifampin is a common phenomenon [8], and in 3 of 6 healthy volunteers the time to C_max_ was greater than 2 h. We observed a reasonable correlation between C_max_ and the urine colorimetric assay (*r* = 0.83), as shown in Fig. 2. For the C_max_ target of 8 mg/L, the area under the ROC curve (Fig. 3) was 0.92 (95 % CI 0.74–1.0). A cutoff of 4 × 10^−2^ absorbance units (AU) had a sensitivity of 92 % (90 % CI 77–100 %) and specificity of 60 % (90 % CI 20–100 %) to detect rifampin serum C_max_ less than 8 mg/L.

### Validation study

**Fig. 4:**
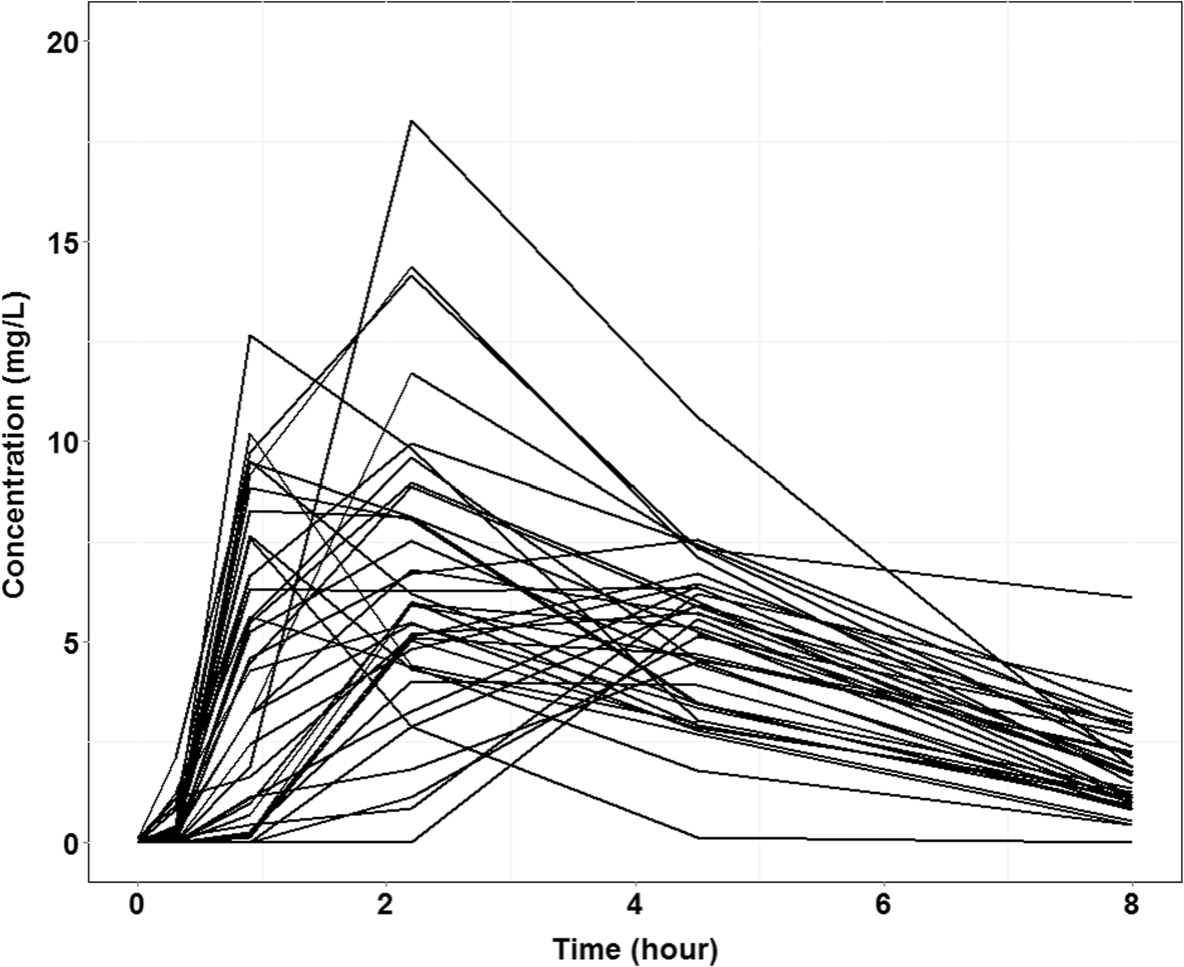
Individual rifampin serum concentration versus time among HIV/TB patients in the validation cohort

Thirty-nine HIV/TB patients completed the intensive PK study and provided a 4-h urine sample for analysis, and all of these patients were included in the validation sample. Baseline demographic and clinical characteristics for these 39 patients are shown in Table 1. Following WHO weight-based dosing guidelines, a single patient was dosed with 300 mg of rifampin, 18 patients were dosed with 450 mg, 17 patients were dosed with 600 mg, and 3 patients were dosed with 750 mg. Individual serum rifampin concentrations versus time are shown in Fig. 4. Based on the observed concentration data, rifampin C_max_ concentrations were below the target of 8 mg/L in 25 of 39 patients (64 %).

**Table 1:** Clinical and demographic characteristics of HIV/TB patients in the validation cohort

Characteristic	HIV/TB patients in the validation cohort (*n* = 39)
Median age (IQR)	32 years (28–44)
Sex	
Male	21 (54 %)
Female	18 (46 %)
Median weight (IQR)	55 kg (50–60)
Median creatinine clearance (IQR)	102 mL/min (91–114)
Past history of pulmonary TB	6 (15 %)
Past history of IPT	5 (13 %)
Past history of any OI	2 (5 %)

**Fig. 5:**
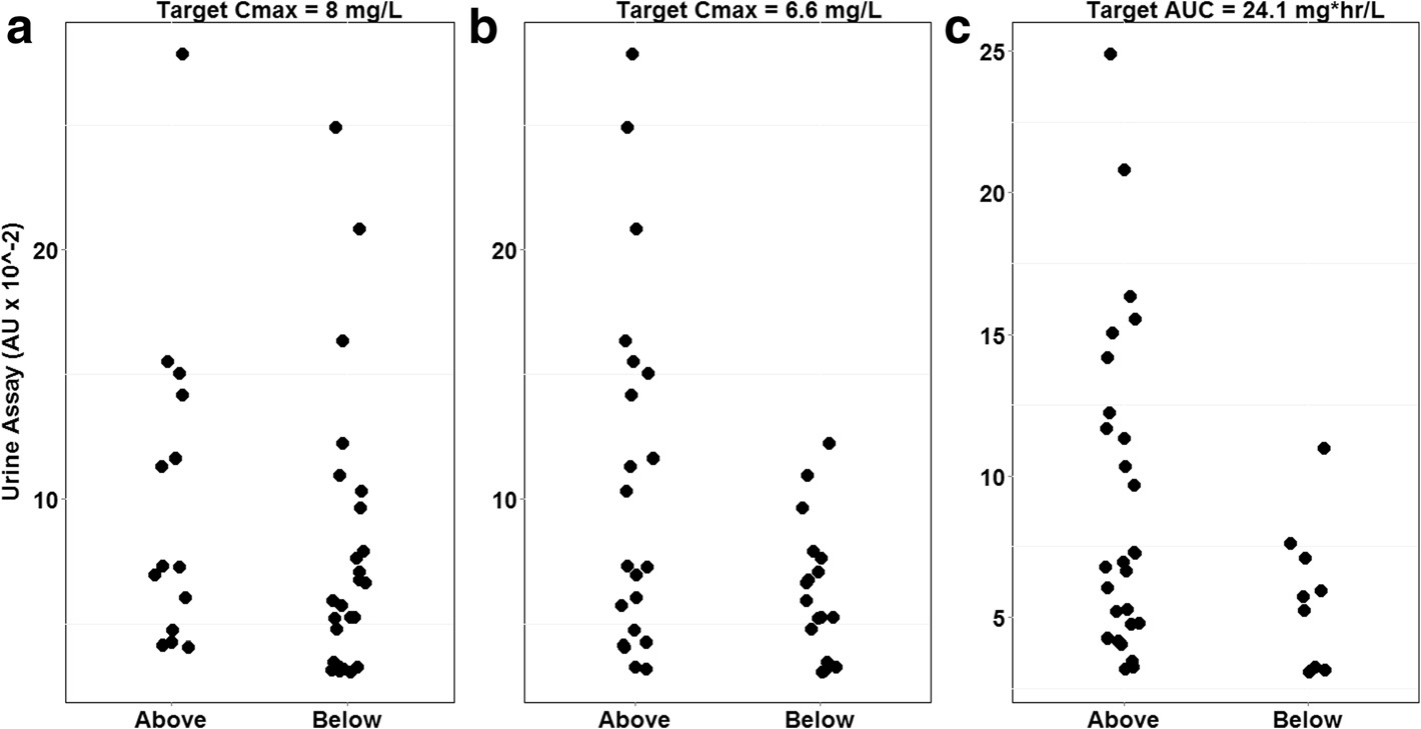
Distribution of urine colorimetric assay among HIV/TB patients based on a serum rifampin target attainment of (a) C _max_ 8mg/L, (b) C _max_ 6.6mg/L, and (c) AUC_0-8_ 24.1mg^*^hr/L

The urine colorimetric assay was poorly correlated with serum C_max_ in the validation cohort (*r* = 0.24). The distribution of the urine colorimetric assay grouped according to serum target attainment is shown in Fig. 5. For the C_max_ target of 8 mg/L (Fig. 5a), the difference in urine colorimetric assay values between groups did not reach statistical significance (*p* = 0.18 by Kruskal-Wallis test). The difference in urine colorimetric assay values between groups was statistically significant (*p* = 0.049) for the secondary C_max_ target of 6.6 mg/L (Fig. 5b), and also reached statistical significance (*p* = 0.02) for the AUC_0–8_ target of 24.1 mg•hour/L (Fig. 5c).

**Fig. 6:**
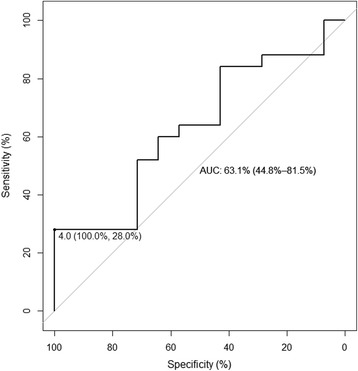
ROC analysis for urine colorimetric assay to detect rifampin C_max_ less than 8 mg/L among HIV/TB patients

The value for the urine colorimetric assay was below the cutoff of 4.0 × 10^−2^ Abs in 7 of 39 patients (18 %), identified among healthy volunteers as predictive of C_max_ less than 8 mg/L. The 2 × 2 table corresponding to this cutoff among HIV/TB patients is shown in Table 2, and the ROC curve is shown in Fig. 6. This cutoff was 100 % specific but poorly sensitive (28 %, 90 % CI 16–44 %). Given that the prevalence of rifampin C_max_ less than 8 mg/L was 64 % in the validation cohort, these diagnostic test characteristics correspond to a positive predictive value of 100 % and a negative predictive value of 44 %. Overall, the urine colorimetric assay demonstrated low diagnostic accuracy for the detection of rifampin C_max_ less than 8 mg/L in the validation sample, with an area under the ROC curve of 0.63 (95 % CI 0.45–0.82).

**Table 2:** Classification table for HIV/TB patients, based on the assay cutoff identified in healthy volunteers

	Rifampin C_max_ less than 8 mg/L	Rifampin C_max_ greater than 8 mg/L
Urine assay less than 4.0 × 10^−2^ Abs	7	0
Urine assay greater than 4.0 × 10^−2^ Abs	18	14

**Fig. 7:**
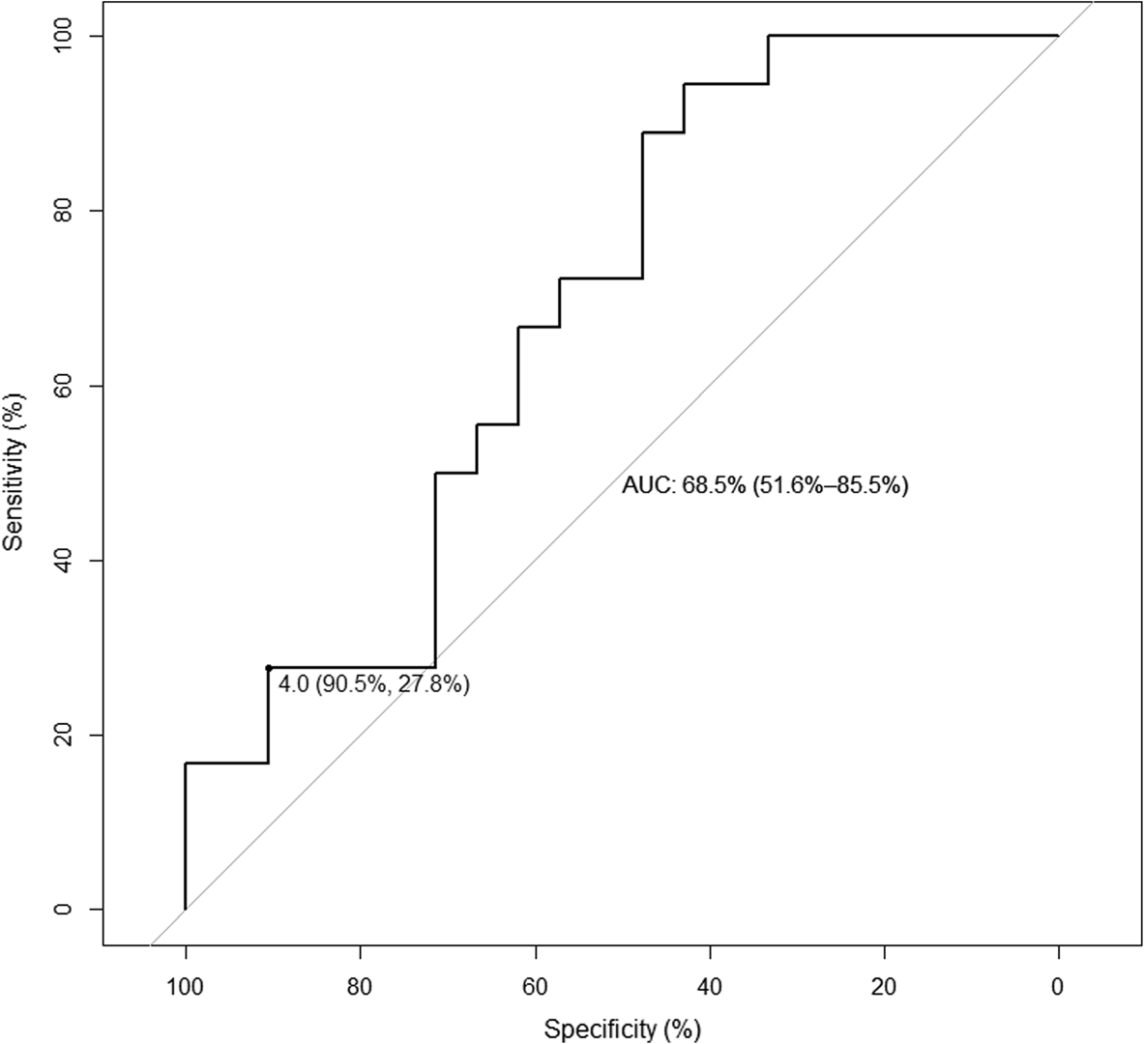
ROC analysis for urine colorimetric assay to detect rifampin C_max_ less than 6.6 mg/L among HIV/TB patients

In secondary analyses, a urine colorimetric assay cutoff of 4.0 x 10^−2^ Abs was 28 % sensitive (90 % CI 11–44 %) and 90 % specific (90 % CI 81–100 %) for identifying patients with a rifampin C_max_ less than 6.6 mg/L, which was the threshold identified as predictive of delayed sputum sterilization among South African TB patients [3]. The area under the ROC curve (Fig. 7) was 0.69 (95 % CI 0.52–0.86). At the optimal threshold identified by Youden’s J statistic, a cutoff of 11.1 × 10^−2^ Abs identified patients with rifampin C_max_ less than 6.6 mg/L with 94 % sensitivity (90 % CI 83–100 %) and 43 % specificity (90 % CI 24–62 %).

**Fig. 8:**
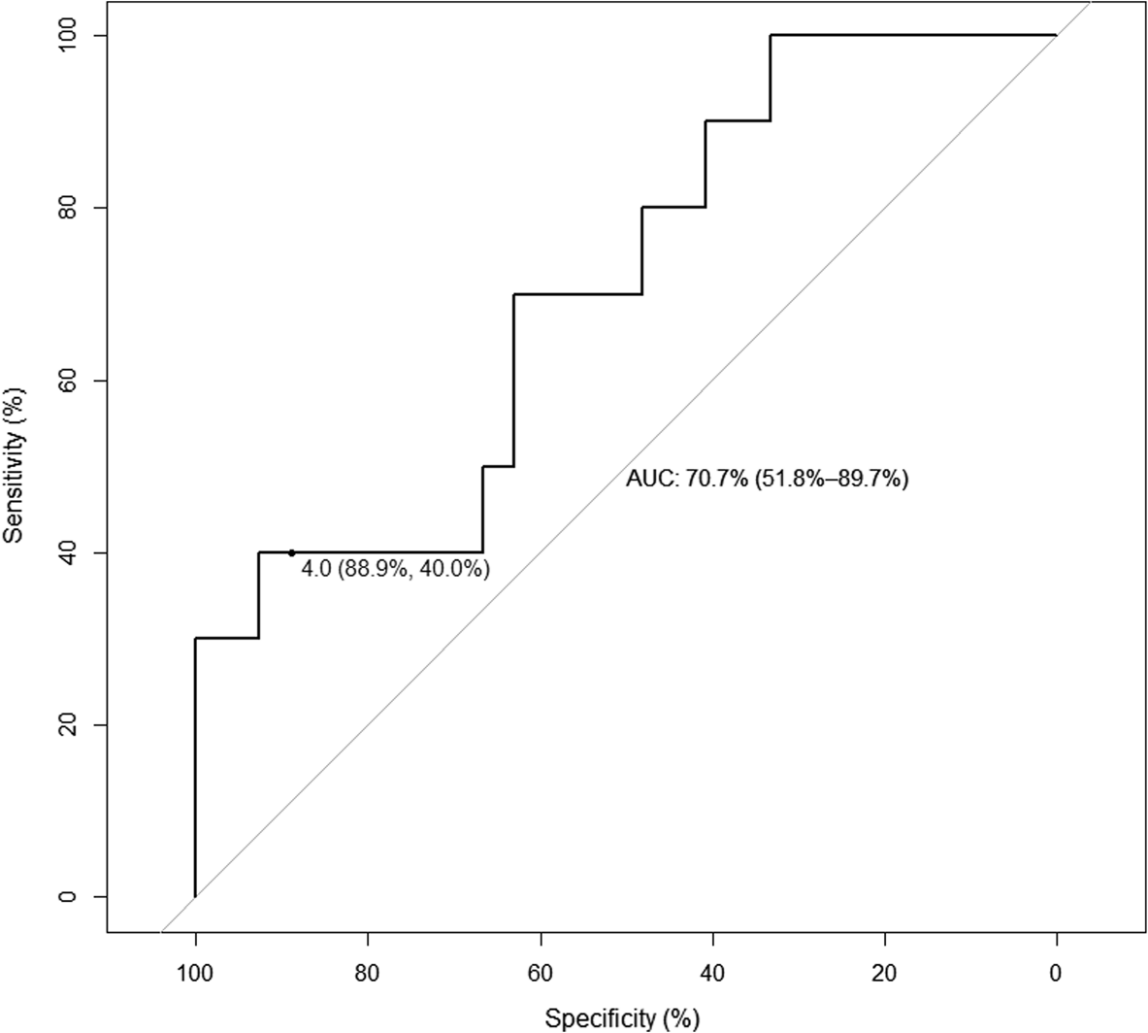
ROC analysis for urine colorimetric assay to detect rifampin AUC_0–8_ less than 24.1 mg•hour/L among HIV/TB patients

The ROC curve corresponding to an AUC_0–8_ target of 24.1 mg•hour/L is shown in Fig. 8. At the cutoff of 4.0 × 10^−2^ Abs, the urine colorimetric assay was 40 % sensitive (90 % CI 20–70 %) and 89 % specific (90 % CI 78–96 %). The summary diagnostic accuracy of the urine colorimetric assay at a target AUC_0–8_ of 24.1 mg•hour/L (area under the ROC curve of 0.71, 95 % CI 0.52–0.90) was similar to targets of 20 mg•hour/L (area under the ROC curve of 0.78, 95 % CI 0.56–1.0) and 30 mg•hour/L (area under the ROC curve of 0.68, 95 % CI 0.50–0.85).

## Discussion

The cost and complexity of current approaches to therapeutic drug monitoring during TB therapy limit widespread use in areas of greatest need, despite the increasing recognition of the role of pharmacokinetic variability in driving treatment outcomes such as delayed sputum sterilization, treatment failure, and the acquisition of new drug resistance [2–6]. The extent of tradeoff between complexity and diagnostic accuracy will determine whether a point-of-care test to estimate anti-TB drug exposure can be a useful addition to the tools of TB clinicians in high-burden settings. There could be additional roles for non-invasive measures of anti-TB drug exposure among pediatric TB populations, where wide pharmacokinetic variability is typically observed, and blood sampling presents an even greater challenge [21].

A simple colorimetric assay of a single urine sample collected 4 h post-dosing performed well in the derivation study with healthy volunteers, but performed less accurately in the validation cohort of HIV/TB patients. The urine colorimetric assay demonstrated similar accuracy for classifying patients based on a C_max_ target of 6.6 mg/L and an AUC_0–8_ target of 24.1 mg•hour; the former has been shown to be predictive of 2-month sputum conversion in TB patients. Nevertheless, test performance characteristics still did not reach the threshold of other commonly used diagnostic tools during the treatment of TB, such as chest radiograph or sputum smear [22, 23].

We selected urine sampling times based on previously published urinary excretion data for rifampin, and chose a time point for evaluation in the validation study based on diagnostic accuracy in healthy subjects. In future work we will employ optimal sampling theory to identify urine sampling schemes that predict serum pharmacokinetics better than a single 4-h timed void [24].

Another limitation of this study is that the development and validation samples arose from different populations. There is important pharmacogenetic variability related to the metabolism and excretion of rifampin, and this variability may contribute to observed differences in the urine assay results between the two groups [25]. Additionally, although the healthy volunteers were dosed with the standard combination regimen (rifampin, isoniazid, pyrazinamide, ethambutol), many of the HIV/TB patients also received additional medications (e.g., cotrimoxazole, fluconazole) as part of their HIV care. The interference of these and other potentially co-administered medications with the urine colorimetric assay for rifampin will require further evaluation.

One strength of our approach was that a priori decisions were made regarding the diagnostic criteria of the optimal cutoff, and the target C_max_ and AUC values to be evaluated. Post-hoc analyses demonstrated that the urine colorimetric assay performed better at lower targets of drug exposure, as defined by C_max_. The use of a borderline or re-testing zone in assay development is likely to improve the positive and negative predictive values for the assay and will be evaluated in subsequent work [26].

The standard rifampin dosing guidelines during anti-TB treatment are increasingly questioned, and a recent dose-finding study of rifampin among TB patients has confirmed the non-linear pharmacokinetics characteristic of rifampin, with disproportionate increases in AUC with increasing dose sizes [27]. The utility of any novel, point-of-care approach will depend on its flexibility to adapt to new evidence for the underlying relationship between drug concentrations and treatment outcomes.

## Conclusion

A urine colorimetric assay for rifampin, conducted on a single urine sample collected 4 h post-dosing, was only modestly accurate in the identification of HIV/TB patients with a rifampin Cmax below 8 mg/L. Future work will focus on refinement of the approach, with the goal of developing a simple, point-of-care, test that could be available for therapeutic drug monitoring during anti-TB therapy in high-burden settings.

## Abbreviations

AU, absorbance unit; AUC, area under the curve; C_max_, maximum concentration; GC, gas chromatography; HIV, human immunodeficiency virus; HPLC, high performance liquid chromatography; PK, pharmacokinetic; ROC, receiver operating characteristic; TB, tuberculosis.
